# Training and Active Case Detection to Prevent Leprosy: Effect on Knowledge, Attitude and Skills of Health Workers on Early Diagnosis of Leprosy in a Leprosy Hotspot District in Ethiopia

**DOI:** 10.3390/tropicalmed9030051

**Published:** 2024-02-23

**Authors:** Ephrem Mamo, Dareskedar Tsehay, Seid Hassen, Solomon Getahun, Addis Mengiste, Beletshachew Tadesse, Tesfaye Tadesse, Mengestu Legesse, Kidist Bobosha

**Affiliations:** 1 Aklilu Lemma Institute of Pathobiology, Addis Ababa University, Addis Ababa P.O. Box 1176, Ethiopia; mengistu.legessed@aau.edu.et; 2 Armauer Hansen Research Institute, Addis Ababa P.O. Box 1005, Ethiopia; dareskedar.tsehay@ahri.gov.et (D.T.); rbpt21@yahoo.com (A.M.); kidist.bobosha@ahri.gov.et (K.B.); 3 Boru Meda Hospital, Dessie P.O. Box 70, Ethiopia; seidhassen09@gmail.com; 4 The Leprosy Mission International Ethiopia, Addis Ababa P.O. Box 30480, Ethiopia; solomong@tlmethiopia.org (S.G.); beletshachewt@tlmethiopia.org (B.T.); 5 Ethiopian National Association of Persons Affected by Leprosy, Addis Ababa P.O. Box 70811, Ethiopia; tesfayehailetadesse@gmail.com

**Keywords:** leprosy, healthcare worker training, early case detection, South Wollo, Ethiopia

## Abstract

Background: Despite all of the efforts, leprosy continues to affect hundreds of thousands of people every year, including children, showing the ongoing transmission of the disease within the population. The transmission of leprosy can be interrupted through an integrated approach that includes active case-finding, contact tracing and capacity building of health workers. Methods: A cross-sectional study design was used to assess the knowledge, attitudes and skills of health workers in the screening and diagnosis of leprosy. One hundred and eighty-one and eighty-eight health care workers participated in the pre-and post-assessment surveys, respectively. Data were collected through interviews and an observational checklist. Frequency tables and graphs were used to describe the study variables, and statistical significance between pre- and post-assessment surveys was declared at *p*-value < 0.5. Result: The percentages of healthcare workers with good knowledge, positive attitudes and skills were 61.2%, 55.6% and 51.7% in the pre-assessment survey and 77.3%, 56.3% and 75.0%, respectively, in the post-assessment survey. There was a significant improvement in the knowledge and skill scores of participants in the post-assessment survey (*p* < 0.01). During the campaign, 3780 index contacts were screened; 570 (15.1%) were diagnosed with skin diseases, and 17 new leprosy cases were diagnosed (case detection rate of 45 per 10,000 contacts). Conclusion: Training improved the knowledge and skills of healthcare workers, and a large number of skin diseases were detected through mass screening and active case findings. Providing training for frontline healthcare workers contributed to the detection of more cases and facilitated early detection of leprosy cases.

## 1. Introduction

Leprosy is a curable infectious disease caused by *Mycobacterium leprae* and is more common in low- and middle-income countries [1,2]. The incubation period is unknown but is likely to last for a period ranging from weeks to years, and the disease develops after the onset of infection. Symptoms include lesions of the skin, peripheral nerves, limbs and eyes and can cause severe disability, stigma and discrimination [2,3,4].

In 2022, 174,087 new cases were reported globally, with a detection rate of 21.8 per million people. The Southeast Asia Region (SEAR) of the WHO accounted for 71.4% of new cases, followed by the Africa Region (AFR) of the WHO (12.6%). Between 2013 and 2022, the number of new cases globally decreased by 19.3% [5]. The COVID-19 pandemic and subsequent control measures that interrupted case finding efforts may have an impact on this figure, and despite the reported decline in the number of new cases detected in the past decades, the proportion of new cases with visible disability at diagnosis (grade 2 disability, G2D) and the proportion of children remained unchanged, indicating a delay in the detection and continued transmission of *M. leprae*. It is estimated that three–four million people live with disabilities due to leprosy. Many of these disabilities can be prevented by early diagnosis and treatment with multidrug therapy (MDT) [6].

According to WHO, Ethiopia had the fifth highest number of reported cases globally in 2022 and the first in Sub Saharan Africa. The number of new cases has decreased slightly over the past ten years, from 4374 in 2013 to 2966 in 2022. In Ethiopia, leprosy management has been integrated into the general health system for two decades, and leprosy patients have mainly been detected through passive case detection [7,8]. However, these figures may be far higher if active case detection is incorporated into the National Tuberculosis and Leprosy Control Program (NTBLCP) [9].

Skin diseases can be clinically screened and diagnosed by appropriately trained individuals with good knowledge, skills and motivation [10]. Healthcare workers’ knowledge and skills in the diagnosis and treatment of leprosy have a substantial impact on case detection delays and leprosy burden. Studies have shown that the limited capacity to diagnose early signs and symptoms of leprosy is an important contributing factor to health system delays [5,6]. Leprosy presents with a wide range of signs and symptoms and is mainly diagnosed clinically, making it challenging for general healthcare providers to notice [11].

Even though the leprosy control program was fully integrated into the general health care system by the end of 2001, Ethiopia has a limited number of health professionals with the skills to diagnose and treat leprosy patients [12,13]. A study conducted in Ethiopia on the performance of general health workers in leprosy diagnosis showed that 86.3 % had poor knowledge of leprosy diagnosis, and only 18.0 % of health workers correctly diagnosed leprosy [12]. Other studies in East Hararghe also identified misdiagnosis as a major problem in the study area, with few leprosy-trained health workers in the districts [14,15]

In most endemic countries, different integrated prevention strategies, such as active case finding, contact tracing and capacity building of health workers through training that aims to reduce the leprosy burden, are in place [16]. These strategies are currently well practiced in many countries [17,18,19] and are considered key interventions for reaching affected patients early and treating them, which eventually helps reduce the transmission of the disease. According to the global leprosy strategy (2021–2030), countries must develop national strategic plans that include intervention for early case detection, reaching the whole population and disability care, and the strategy also calls for accelerating action to reach the goal of zero leprosy [6]. For countries to progress towards zero leprosy, active case detection and capacity building of health workers are included as pillars. This study aimed to assess the effect of training on frontline health workers’ knowledge, attitudes and skills in diagnosing leprosy cases through active case detection campaigns.

## 2. Methods and Analysis

### 2.1. Study Setting

This study was conducted in the South Wollo zone of the Amhara region, Ethiopia. South Wollo is a high-spot zone for leprosy in the Amhara Region. Among the 20 districts in the South Wollo zone, 15 were hotspots for leprosy, with child leprosy cases >5% and G2D > 10%. The Amhara Sayint, Mekdela, Tenta and Mehal Sayint districts are hotspot districts as reported in the leprosy mapping report [20].

### 2.2. Study Design

A cross-sectional study design was used to assess the knowledge, attitudes and skills of health workers in screening and diagnosing leprosy (pre- and post-assessment).

### 2.3. Study Population

Clinical nurses, health officers and medical doctors working in outpatient departments (OPDs) of selected districts participated in the study. One hundred and eighty-one and eighty-eight healthcare workers participated in the pre- and post-assessment surveys, respectively.

The main reason for the reduced number of healthcare workers in the post-assessment survey was the high staff turnover rate in the study area, which were mostly brought on by security concerns as a result of conflict in northern Ethiopia, and also to see the effect of integrating training with mass screening, we only considered health workers involved in the mass screening in post-assessment survey.

### 2.4. Training of Health Workers

Five days of training were provided to frontline healthcare workers working at the OPD. Training focused on improving the capacity for the identification, diagnosis and management of leprosy patients. The training was interactive and skill/practice-based, enabling participants to screen and confirm leprosy cases. The training covered the epidemiology of leprosy, basics of leprosy or mode of transmission, leprosy case-finding strategies, identification and evaluation of patients to diagnose leprosy: clinical history, physical examination (skin sensation testing and examination of the nerves), disability grading in leprosy, case definition, classification and treatment of leprosy, monitoring of treatment and follow-up, referral of leprosy patients for special care, complications of leprosy and its management, reaction management, disability reduction including self-care practice, differential diagnosis, stigma and discrimination.

### 2.5. Active Case Detection Campaign

Following the training, an active case detection campaign was conducted. The campaign was organized at the community level (at the (sub-) village/neighborhood level of the index patient) in close collaboration with community leaders and local organizations, with on average around 15–20 households (household contacts and neighbors were screened for leprosy) per index [21,22]. This campaign was conducted in schools and marketplaces. Trained healthcare workers, leprosy experts and dermatologist are involved in mass screening campaigns.

An integrated skin disease approach was used for contact screening. Integration in this context involved screening of two or more skin diseases simultaneously in the community, according to the WHO/NTD guideline [23]. This is important to overcome negative factors such as perceived social stigma and discrimination that may limit participation when focusing on one stigmatizing disease such as leprosy [24,25,26,27]. If leprosy was confirmed, MDT treatment was preferably started on the same day after referral to the nearest health facility, and further treatment was administered in the health center according to the national guidelines.

### 2.6. Data Collection and Analysis

Three days of training were provided to the data collectors and supervisors regarding the data collection tool and procedures. Interviews with healthcare workers were conducted using a pre-tested, structured questionnaire. The questionnaire included socio-demographic data, a knowledge and attitude section comprising 8 items, and a skills section that contained 10 items. The skills were assessed using a structured, predefined observational checklist. The skills of each selected healthcare worker were assessed while they were screening a suspected leprosy patient at their respective health facilities prior to the training and after mass screening campaign. If there was no suspected leprosy at the time of the visit, one of the data collectors imitated suspected leprosy and was evaluated by the health workers. A trained field researcher conducted interviews and observations. The data were checked, cleaned and double-entered into the Research Electronic Data Capture (REDCap) database and exported to SPSS version 25 for analysis. To determine knowledge, attitude and skills, each summative score was converted to a binary variable using the mean as the cut-off point: knowledge (good or poor), attitude (negative or positive) and skills (good or poor), respectively [14,28]. The mean and 95% confidence interval (CI) were used to describe the KAP score, and the paired t-test was used to detect differences between the pre-and post-assessment scores, with significance declared at *p* < 0.05. The study variables were described using descriptive statistics such as frequency tables and graphs.

## 3. Result

### 3.1. Socio Demographic Characteristics of the Respondents

A total of 181 participants, with a mean age of 27.7years (SD ± 5.3), were included in the pre-assessment survey (Table 1). Most participants were male 126 (68.9%) and nearly one third 56 (30.9%) were nurses. Regarding previous training, 162 (88.5%) health workers had neither received leprosy training before nor worked in the field of leprosy 151 (82.5%). Of those working in the field of leprosy, 15 (48.4%) were involved in screening and diagnosis, while 4 (12.9%) were involved in the prevention of disabilities after treatment, including referral of leprosy patients for special care, reaction management and teaching patients about self-care practice.

Eighty-eight healthcare workers who took part in the mass screening were enrolled in the post-assessment survey. The mean age was 28.5 years (SD ± 5.8). Almost half (42 (47.7%)) were health officers, whereas only 3 were medical doctors. During the post-assessment survey, 29 health care workers (33.0%) were directly involved in the field of leprosy. Of these, 16 (55.2%) were involved in screening and diagnosis, while 3 (10.3%) were involved in the prevention of disabilities after treatment.

### 3.2. Knowledge Level of Health Care Workers about Leprosy

**Figure 1:**
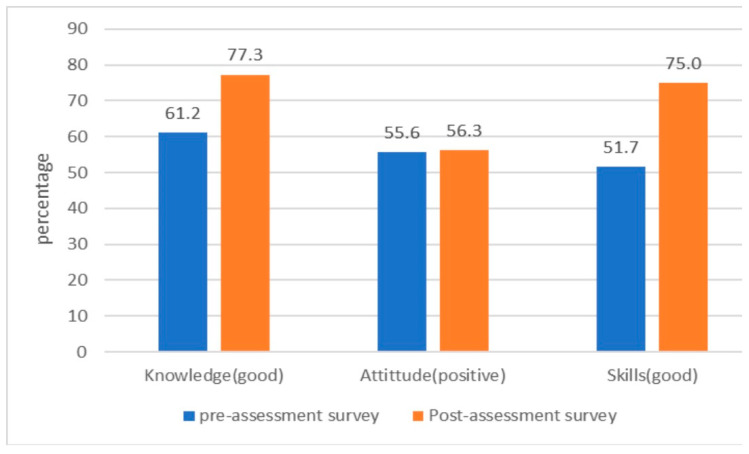
Knowledge, attitude and skills of health care workers on screening and diagnosis of leprosy.

Differences were observed in the responses to the knowledge assessment questions in the pre- and post-assessment surveys. On the questions regarding who could get leprosy (72.4% vs. 92%), the possible complications of leprosy (56.9% vs. 80.7%), correct statements about leprosy (71.7% vs. 95.5%), contagiousness of leprosy patients halfway through treatment (58.8% vs. 76.1%) and the duration of leprosy treatment (45.4% vs. 92%) (Table 2). At pre-assessment and post-assessment surveys, the percentages of healthcare workers with good knowledge were 61.2% and 77.3%, respectively (Figure 1). The mean knowledge scores were 4.7 (95%CI: 4.4, 4.9) and 9.3 (95%CI: 9.0, 9.6) in the pre- and post-assessment survey, respectively, showing significant improvement in knowledge score between pre-assessment and post-assessment survey (*p* < 0.01) (Table 3).

### 3.3. Attitude of Health Care Workers towards Leprosy

The results of the respondents’ attitudes towards leprosy are shown in Table 4. In both the pre-assessment and post-assessment surveys, the majority of participants believed that leprosy was a major public health problem in our country (56.7% and 57.9%, respectively). The discrepancy was that there were no negative responses in the post-assessment survey when asked if they were happy to diagnose and treat leprosy compared with the few respondents in the pre-assessment survey (14 (7.9%)). In the post-assessment survey, about half (53.4%) of the respondents disagreed with the question regarding the possibility of contracting leprosy while treating ex-leprosy patients with deformities, which was higher than that in the pre-assessment survey (31.7%).

When comparing the pre-assessment and post-assessment surveys, little to no difference was seen in their responses to questions about their perception on increased risk of contracting the disease while managing a leprosy patient; 16.4% in pre- and 19.3% in post-assessment survey strongly disagreed, and when isolating leprosy patients from admitted patients, 10.7% of health care worker in pre-and 11.4% in post-assessment survey strongly disagreed.

There was no significant difference in the mean attitude score of respondents after training (*p* > 0.05), and only a 0.7% increase in the proportion of respondents with positive attitudes was observed when comparing the pre- and post-assessment surveys (55.6% vs. 56.3%, respectively) (Figure 1)

### 3.4. Skill of Health Care Workers about Leprosy

As shown in Table 5, respondents’ pre- and post-assessment skills improved in all areas. Healthcare workers had a considerable improvement in examining patients with skin color change on the body (41.4% vs. 76.1%), if loss of sensation was present (31.0% vs. 77.3%), in describing the typical skin lesion in leprosy (34.5% vs. 58.0%), and in instructing the patients when and how to respond to sensory exams (24.1% vs. 47.7%), identifying and properly examining hand muscles (3.4% vs. 47.7%) and correctly diagnosing and classifying leprosy cases (6.9% vs. 46.6%) (Table 5). The proportion of healthcare workers with good skills increased by 23.3% in the pre-assessment survey (51.7%) (Figure 1). A statistically significant difference was observed between pre-assessment and post-assessment mean skill scores (*p* < 0.05) (Table 3).

### 3.5. Mass Screening

Following the training, a mass-screening campaign was conducted in the four districts. Eighty-eight health workers who received training and were available during the campaign were involved in the mass screening. On average, 20 households and 100 contacts (household contacts and neighbors) who lived in the surrounding area of the index case house were screened for leprosy and other skin diseases. Patients diagnosed with leprosy within six months prior to the study were included as index cases. In addition to the house-to-house survey, participants were screened at schools, public gatherings, churches and mosques.

The results of the campaign showed that 67 index cases were included in the mass screening, 617 houses were visited, and 3780 contacts were screened, and among screened contacts, 570 (15.1%) were diagnosed with skin disease and 17 (45 per 10,000 contacts) were diagnosed with leprosy. Of the confirmed cases, two (11.7%) were under 15 years of age, three (17.6%) were women, fourteen (82.4%) were multibacillary and three (17.6%) had G2D (Table 6).

## 4. Discussion

The majority of participants in both the pre- and post-assessment surveys were male professional nurses. Most healthcare workers who participated in the pre-assessment survey had neither received leprosy training nor worked in the field of leprosy. The proportion of healthcare workers who had good knowledge in the pre-assessment survey was low, but after training and participation in mass screening, the proportion increased by 16.1%. The overall knowledge score showed a significant improvement in the health workers towards the diagnosis and management of leprosy. Consistent with our findings, a study on general health workers in Ethiopia found a substantial increase in the proportion of healthcare workers who had good knowledge after receiving 3 days of training [28]. Similarly, there was an improvement in knowledge and confidence levels among family medicine physicians in Malaysia after a 3-day lecture [29]. Other studies carried out in India, Bangladesh and Malaysia have shown that training played a key role in improving their knowledge [30,31,32]. Abeje et al. also reported that good knowledge was associated with training [12].

Another notable finding of this study was the significant improvement in healthcare workers’ skills. The proportion of healthcare workers with good skills increased by 25% in the pre-assessment survey. This difference is attributed to the intervention of training, on-site supervision and support of healthcare workers during mass screening [33]. These results are in line with those of Dellar et al. and Tsehaynesh et al., who demonstrated that training greatly enhanced healthcare workers’ skills in diagnosing leprosy [17,28].

Our study found no significant improvement in health workers’ attitudes after the training. This may be because attitude changes require more time. The findings of this study are in line with those of previous studies that demonstrated that a training intervention was linked to little or no change in terms of improving healthcare professionals’ attitudes toward leprosy [28,30]. The training content also has an effect in the change of health workers’ attitude. Similar to earlier studies, the training materials in this study are primarily focused on the knowledge and skills of healthcare workers.

In this study, many skin diseases, including leprosy, were detected during the mass screening. Similarly, a study conducted in Benin on an integrated approach in the control and management of skin-neglected tropical diseases showed that a high number of skin diseases were diagnosed after training in the integration approach of skin disease screening [34]. Studies conducted on leprosy in high-burden rural sites in Ethiopia and Sri Lanka have shown that training combined with mass screening has a sound effect on early case detection and contact tracing [17,35].

Timely diagnosis with adequate treatment and follow-up minimizes the risk of developing complications and permanent disabilities in patients with leprosy [36,37,38]. A key proximal factor contributing to the case detection delay is misdiagnosis. As leprosy symptoms vary widely, it can be difficult for general healthcare providers to identify the disease [39]. If it is not diagnosed immediately, individuals with leprosy may require more visits to health care providers. Longer detection delays can also be caused by incorrect diagnosis [40]. Hence, capacity building of healthcare professionals is a vital factor in the screening, diagnosis and treatment of patients with leprosy. Our study shows that an integrated training program combined with an active case detection approach can improve frontline healthcare workers’ knowledge and skills in leprosy diagnosis and treatment, which in turn increases the rate of new case detection.

A key strength of this study is that it was conducted by skilled data collectors who were trained in how to use the questionnaire. Dermatologist and leprosy experts also participated in training and active case detection. An integrated skin disease approach was employed for contact screening. This is important to overcome negative factors that could prevent people from participating when focusing on one stigmatizing disease such as leprosy. The major limitation of this study is the high turnover of healthcare workers, which resulted in a reduced number of healthcare workers who participated in the post-assessment survey. As our training tool are mostly focused on knowledge and skills, the same as in prior studies, an additional study is recommended to be conducted after modifying the training materials.

## 5. Conclusions

The knowledge and skills related to early leprosy screening and diagnosis among the healthcare workers assessed in the study area were improved by training and mass screening. Combining active case-finding and training for healthcare workers is expected to lead to more and earlier detection of leprosy cases. The integrated approach to skin diseases allows the detection of a large number of skin diseases in a single mass screening and avoids stigma.

## Figures and Tables

**Table 1:** Socio demographic characteristics of the respondents.

Variables	Category	Frequency (%)	Frequency (%)
		Pre-Assessment (n = 181)	Post-Assessment (n = 88)
Sex	Male	126 (68.9%)	75 (85.2%)
	Female	55 (30.1%)	13 (14.8%)
Occupation	Nurse diploma	87 (28.2%)	25 (28.4%)
	Nurse degree	56 (30.9%)	13 (14.8%)
	Health officer	37 (20.4%)	42 (47.7%)
	MD (General Practitioner)	29 (16.0%)	3 (3.4%)
	Others	8 (4.4%)	5 (5.7%)
Have you received Leprosy training before?	Yes	19 (10.4%)	88 (100%)
	No	162 (88.5%)	0
Have you worked in the field of leprosy?	Yes	31 (16.9%)	29 (33.0%)
	No	151 (82.5%)	59 (67.0%)
Which field of leprosy care are you involved or active in?	Screening and diagnosis	15 (48.4%)	16 (55.2%)
	Treatment	7 (22.6%)	6 (20.7%)
	Care and follow up	5 (16.1%)	4 (13.8%)
	Prevention of disabilities	4 (12.9%)	3 10.3%)

**Table 2:** Questions that assessed knowledge level of health workers regarding leprosy.

S. No	Knowledge Questions	Pre-Assessment (n = 181)		Post-Assessment (n = 88)	
		1 = Correct	0 = Incorrect	1 = Correct	0 = Incorrect
1.	Who can get leprosy?	131 (72.4%)	50 (27.6%)	81 (92.0%)	7 (8.0%)
2.	What causes leprosy?	161 (89.4%)	19 (10.6%)	81 (92.0%)	7 (8.0%)
3.	What is an early symptom of leprosy?	117 (64.6%)	64 (35.4%)	58 (65.9%)	30 (34.1%)
4.	What is a possible complication of leprosy?	103 (56.9%)	78 (43.1%)	71 (80.7%)	17 (19.3%)
5.	Which statement about leprosy is correct?	129 (71.7%)	51 (28.3%)	84 (95.5%)	4 (4.5%)
6.	Can leprosy be treated?	129 (71.3%)	52 (28.7%)	64 (72.7%)	24 (27.3%)
7.	A patient is half-way through his medication courses against leprosy, and he/she took the medication properly, is he/she still contagious?	107 (58.8%)	75 (41.2%)	67 (76.1%)	21 (23.9%)
8.	What is the duration of leprosy treatment of PB (Paucibacillary) leprosy?	81 (45.4%)	100 (54.6%)	81 (92%)	7 (8%)

**Table 3:** Mean score of knowledge, attitude and skills of health workers on screening and diagnosis of leprosy.

Variable	Pre-Assessment					Post-Assessment			
	n	Mean Score	95% CI for Mean		n	Mean Score	95% CI for Mean		*p* Value
			Lower Bound	Upper Bound			Lower	Upper	
Knowledge score	181	4.7	4.4	4.9	88	9.3	9.0	9.6	0.01
Attitude score	181	29.5	28.8	30.2	88	30.8	29.8	31.8	0.08
Skill score	29	22.0	19.6	24.4	88	34.4	33.3	35.5	0.01

**Table 4:** Question used to assess the attitude level of health workers toward leprosy.

S. No	Attitude Questions (n = 178) Pre-Assessment	Pre-Assessment Survey Frequency (Percent)				Post-Assessment Survey Frequency (Percent)					
		Strongly Disagree	Disagree	Neutral	Agree	Strongly Agree	Strongly Disagree	Disagree	Neutral	Agree	Strongly Agree
1	Leprosy is a major public health problem in our country	5 (2.8%)	15 (8.4%)	12 (6.7%)	86 (48.3%)	60 (56.7%)	2 (2.3%)	4 (4.5%)	1 (1.1%)	30 (34.1%)	51 (57.9%)
2	I am happy to diagnose and treat leprosy cases	5 (2.8%)	9 (5.1%)	2 (1.1%)	61 (34.3%)	101 (35.9%)	0	0	0	15 (17%)	73 (83%)
3	It is possible to manage leprosy in the general healthcare like any diseases	18 (10.1%)	27 (15.2%)	14 (7.9%)	60 (33.7%)	59 (33.1%)	3 (3.4%)	9 (10.2%)	4 (4.5%)	30 (34.1%)	42 (47.7%)
4	There is a high risk of contracting the disease while managing a leprosy patient	29 (16.4%)	46 (26.0%)	19 (10.7%)	61 (34.5%)	22 (12.4%)	17 (19.3%)	24 (27.3%)	9 (10.2%)	23 (26.1%)	15 (17.0%)
5	It is good to isolate leprosy in-patients from admitted patients	19 (10.7%)	32 (18.0%)	15 (8.4%)	70 (39.3%)	42 (23.6%)	10 (11.4%)	18 (20.5%)	3 (3.4%)	34 (38.6%)	23 (26.1%)
6	It is very important to trace leprosy patients who don’t come for treatment	20 (11.2%)	21 (11.8%)	14 (7.9%)	58 (32.6%)	65 (36.5%)	5 (5.7%)	9 (10.2%)	1 (1.1%)	19 (21.6%)	54 (61.4%)
7	It is very important to trace leprosy family contacts	11 (6.2%)	11 (6.2%)	15 (8.4%)	5 (2.8%)	64 (36%)	6 (6.8%)	10 (11.4%)	0	16 (18.2%)	56 (63.6%)
8	There is a possibility of contracting leprosy while treating ex-leprosy patient with deformities.	18 (10.2%)	18 (10.2%)	38 (21.5%)	28 (15.8%)	75 (42.4%)	20 (22.7%)	27 (30.7%)	7 (8%)	19 (21.6%)	15 (17.0%)

**Table 5:** Questions to assess the skills level of health workers regarding leprosy.

S. No	Skill Questions Pre-Assessment (n = 29)	Pre-Assessment Survey Frequency (Percent)				Post-Assessment Survey Frequency (Percent)			
		Not Done	Done Incorrectly	Done Moderately	Done Perfectly	Not Done	Done Incorrectly	Done Moderately	Done Perfectly
1.	If they have any skin color change in the body	2 (6.9%)	4 (13.8%)	11 (37.9%)	12 (41.4%)	4 (4.5%)	1 (1.1%)	16 (18.2%)	67 (76.1%)
2.	If they have loss of sensation or burning sensation on the skin	2 (6.9%)	3 (10.3%)	15 (51.7%)	9 (31.0%)	2 (2.3%)	1 (1.1%)	17 (19.3%)	68 (77.3%)
3.	Describe the typical skin lesions in leprosy	3 (10.3%)	6 (20.7%)	10 (34.5%)	10 (34.5%)	0	4 (4.5%)	33 (37.5%)	51 (58.0%)
4.	Instruct the patient when and how to respond for skin examination	6 (20.7%)	8 (27.6%)	8 (27.6%)	7 (24.1%)	2 (2.3%)	10 (11.4%)	16 (18.2%)	60 (68.2%)
5.	Examine the skin lesion with cotton	5 (17.2%)	6 (20.7%)	8 (27.6%)	10 (34.5%)	0	5 (5.7%)	20 (22.7%)	63 (71.6%)
6.	Instruct the patient when and how to respond for sensory exam	16 (55.2%)	6 (20.7%)	5 (17.2%)	2 (6.9%)	3 (3.4%)	12 (13.6%)	31 (35.2%)	42 (47.7%)
7.	Identify and properly examine muscles of the hand	17 (62.1%)	10 (34.5%)	0	1 (3.4%)	5 (5.7%)	14 (15.9%)	27 (30.7%)	42 (47.7%)
8	Identify and properly examine muscles of the feet	15 (51.7%)	9 (31.0%)	2 (6.9%)	3 (10.3%)	5 (5.7%)	12 (13.6%)	27 (30.7%)	40 (50.0%)
9	Correctly diagnose and classify a case of leprosy	16 (55.2%)	7 (24.1%)	4 (13.7%)	2 (6.9%)	1 (1.1%)	13 (14.8%)	33 (37.5%)	41 (46.6%)
10	Accurately grade the disability status of leprosy	17 (58.6%)	7 (24.1%)	3 (10.3%)	2 (6.9%)	1 (1.1%)	16 (18.2%)	28 (31.8%)	43 (48.9%)

**Table 6:** Mass screening campaign conducted in South Wollo, 2022.

Variable	Frequency	Percentage
Number of index patients included	67	
Number of community contacts screened	3780	
Number of skin diseases diagnosed	570	15.1%
Number of confirmed leprosy cases	17 (45/10,000 contact)	
Sex		
Male	14	82.4%
Female	3	17.6%
Age group		
<15 years	2	11.8%
≥15 years	15	88.2%
Leprosy subtype		
PB	3	17.6%
MB	14	82.4%
Disability grade		
None	10	58.8%
G1D	4	23.5%
G2D	3	17.7%

Abbreviations: PB: paucibacillary; MB: multibacillary; G1D: grade-1 disability; G2D: grade-2 disability.

## Data Availability

The results of this research were extracted from the data gathered and analyzed based on the stated methods and materials. The original data supporting this finding are available upon request.

## Abbreviations

AHRI: Armauer Hansen Research Institute; AAERC: AHRI ALERT Ethics Review Committee; G1D: grade-one disability; G2D: grade-two disability; MB: multi bacillary; MDT: multi drug treatment; PB: paucibacillary; PHC: primary health care.
